# Early-switch versus late-switch in patients with diabetic macular edema: a cost-effectiveness study

**DOI:** 10.1007/s00417-022-05892-3

**Published:** 2022-11-12

**Authors:** José M. Ruiz-Moreno, Jorge Ruiz-Medrano

**Affiliations:** 1 Puerta de Hierro-Majadahonda University Hospital, 38 Melendez Valdes, 28015 Madrid, Spain; 2 Department of Ophthalmology, Castilla La Mancha University, Albacete, Spain; 3 Miranza, Spain

**Keywords:** Diabetic macular edema, Switching, Dexamethasone intravitreal implant, VEGF inhibitors, Cost-effectiveness ratio, Incremental cost effectiveness ratio

## Abstract

**Background:**

To evaluate the cost-effectiveness of early- versus late-switch to the intravitreal-dexamethasone implant (DEX-i) in patients with diabetic macular edema (DME) who did not adequately respond to vascular endothelial growth factor inhibitors (anti-VEGF).

**Methods:**

Retrospective analysis of a multicenter Clinical Data Registry. The registry included DME eyes who received 3 intravitreal anti-VEGF injections (early-switch) or > 3 intravitreal anti-VEGF injections (late-switch) before switching to DEX-i injections. The primary outcome was to estimate the incremental cost needed to obtain a best-corrected visual acuity (BCVA) improvement ≥ 0.1 or a central-retinal thickness CRT ≤ 250 μm.

**Results:**

The analysis included 108 eyes, 32 (29.6%) and 76 (70.4%) in the early- and late-switch groups, respectively. Early-switch strategy was associated with a cost saving of €3,057.8; 95% CI: €2,406.4–3,928.4, *p* < 0.0001). Regarding incremental-cost-effectiveness ratio, late-switch group was associated with an incremental cost of €25,735.2 and €13,533.2 for achieving a BCVA improvement ≥ 0.1 at month 12 and at any of the time-point measured, respectively. At month 12, 38 (35.2%) eyes achieved a BCVA improvement ≥ 0.1. At month 12, 52 (48.1) eyes had achieved a CRT ≤ 250 micron. As compared to baseline, the mean (95% CI) CRT reduction was − 163.1 (− 212.5 to − 113.7) µm and − 161.6 (− 183.8 to − 139.3) µm in the early-switch and late-switch groups, respectively, *p* = 0.9463.

**Conclusions:**

In DME eyes, who did not adequately respond to anti-VEGF, switching to DEX-i at early stages (after the first 3-monthly injections) was found to be more cost-effective than extending the treatment to 6-monthly injections of anti-VEGF.


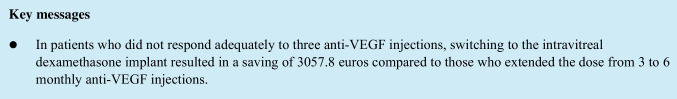


## Introduction 

Sociodemographic changes in the population have posed important challenges for health systems. As worldwide elderly population continues to increase and get older, healthcare costs are expected to rise even further. Health systems, therefore, must be increasingly efficient for affording the growing demand of patients affected by chronic diseases [1].

The prevalence of diabetes mellitus (DM), particularly type 2 DM, has been increasing rapidly over the past few decades [2, 3]. Based on data from the International Diabetes Federation, the global prevalence of diabetes has been estimated to be 12.2% (783.2 million people) by 2045 [4], which represents a health expenditure of approximately 845 billion dollars [5].

Diabetic retinopathy (DR) is the leading legal blinding eye disease for working-aged people (accounting for about 2.6% of all vision losses), and diabetic macular edema (DME) secondary to DR is the direct cause of visual impairment [6–8].

Vascular endothelial growth factor (VEGF) inhibitors (anti-VEGF) are commonly used as a first-line therapy for DME [9, 10]. However, as many as 30–50% of patients do not respond to anti‐VEGF treatment adequately [11, 12]. Moreover, eyes with a poor response to ranibizumab (those gaining < 5 letters after 3-monthly intravitreal ranibizumab injections) usually do not improve further with continuing in ranibizumab treatment [13] and extending the dose to 24 weeks was not associated with better functional or anatomic outcomes [14].

From a clinical point of view, the relevance of these findings critically depends on whether patients who did not adequately respond to anti-VEGF therapies could benefit from other therapies, especially considering that long-standing DME can permanently damage the retina, which might limit the functional recovery [15].

Intravitreal dexamethasone implant (DEX-i) has proven to have a beneficial impact on both anatomic and functional clinical outcomes since it is capable of acting both on inflammatory and on vasogenic mediators [16–18]. Additionally, patients who did not adequately respond to anti‐VEGF treatment should be switched to DEX-i as soon as possible, and preferably after 3 doses of anti-VEGF [19, 20].

Because DME treatment entails a high cost, the suitability of new treatments must be established on the basis of the patient’s benefit.

Despite the clinical, social, and economic relevance of this therapy area, data evaluating the cost effectiveness of DEX-i in DME are limited [21–24].

In a previous paper, we assessed the clinical and economic consequences of an extension of initial anti-VEGF treatment from 3 to 6 monthly injections in patients with persistent central-involved DME and visual impairment based on the findings of the post hoc analyses of the DRCR.net Protocol T clinical trial. The results of the study showed that for the total number of patients treated, an average of €7927.02 per additional responder patient would be necessary [25].

This study aimed to evaluate the cost-effectiveness of early- versus late-switch to the DEX-i in DME patients who did not adequately respond to anti-VEGF therapies. This study is based on the findings of our previous paper comparing the clinical outcomes of early versus late switch in eyes who did not adequately respond to anti-VEGF therapies and underwent a DEX-I [20].

## Methods

Retrospective analysis of a multicenter Clinical Data Registry

A cost-consequence model was developed in Microsoft Excel following recommendations for economic evaluation of health technologies [26]. The model compared the economic implications of switching non-responder patients to 3-monthly intravitreal injections of anti-VEGF to DEX-i (early-switch) to those of extending the anti-VEGF initial treatment from 3 to 6-monthly injections (late-switch).

This analysis was carried out from a hospital pharmacy perspective in which only pharmaceutical treatment costs were considered. Other direct medical costs such as costs of administration were not included as they were beyond the scope of this analysis. The anti-VEGF treatment options included in the model were aflibercept, ranibizumab, and bevacizumab. To estimate the pharmaceutical costs, official ex-factory unit prices were obtained from the General Council of Provincial Pharmacy Chambers database [27]: aflibercept 40 mg/ml (€578.76 per vial), ranibizumab 10 mg/ml (€619.75 per vial) and bevacizumab 25 mg/ml (€262.0 per vial; 4 ml presentation). All the costs included in the analysis reflected the value in euros for the year 2021.

This study analyzed the cost and consequences of the continuation until month 12, of monthly treatment of DME patients with anti-VEGF from month 3 to month 6 versus switching to DEX-i at month 3 in non-responder patients according to the results published in our previous study [20].

### Definitions

“Not-adequately” respond to anti-VEGF was defined if after 3 anti-VEGF injections (either ranibizumab, bevacizumab, or aflibercept), there was (1) no improvement in best corrected visual acuity (BCVA) and/or (2) a central retinal thickness (CRT) reduction < 20% and/or (3) recurrence of DME despite monthly anti-VEGF injections and/or (4) similar BCVA but worsening of DME; and/or (5) decreased in BCVA and a CRT thickening [25].

Early-switch: Eyes who did not respond adequately to three-monthly injections of anti-VEGF and were switched to DEX-i.

Late-switch: Eyes who did not respond adequately to three monthly injections of anti-VEGF and received an extending initial treatment of three-monthly anti-VEGF injections up to 6 months.

### Calculations

Cost-effectiveness ratio (CER) was calculated as per cost/outcome formula. CER was once calculated by considering the numerical difference of outcomes.

Incremental cost-effectiveness ratio (ICER) was calculated by the numerical difference of outcomes between the early-switch and late-switch groups as the following formula: ICER = (cost in early-switch–cost in late-switch group)/(effectiveness in early-switch group–effectiveness in late switch group).

### Outcomes

The primary outcome was to estimate the incremental cost needed to obtain an additional response at month 12 (improvement in BCVA ≥ 0.1 or reduction in CRT ≤ 250 μm) in patients who maintained anti-VEGF therapy versus those who switched to DEX-i.

The secondary outcomes included the cost of achieving different BCVA, changes in BCVA, changes in CRT and subfoveal choroidal thickness (SCT), and the probability of achieving different BCVA.

### Statistical analysis

A standard statistical analysis was performed using the MedCalc® Statistical Software version 20.110 (MedCalc Software Ltd, Ostend, Belgium; https://www.medcalc.org; 2022).

Descriptive statistics number (percentage), mean [standard deviation (SD)], mean [95% confidence interval (95% CI)], or median [Interquartile range (IqR)] were used, as appropriate.

Distribution of quantitative variables was assessed using the D’Agostino-Pearson test.

The Mann–Whitney *U* test was used in the evaluation of the baseline quantitative variables and the total costs between early-switch and late-switch groups. A repeated measures ANOVA or a Friedman’s two-way analysis test as appropriate were used to assess changes in BCVA and CRT within the groups throughout the study.

The Cochran-Mantel–Haenszel test was used to assess the probability of achieving a BCVA improvement ≥ 0.1 (at any time-point measured and at month 12) or ≥ 0.2 (at any time-point measured and at month 12) with study group as grouping variable and type of DME as factor variable.

## Results

### Baseline demographic and clinical characteristics

One hundred and eight eyes were included in the analysis, 32 (29.6%) in the early-switch group and 76 (70.4%) in the late-switch group.

In the overall study, sample mean age was 67.6 ± 8.9 years and 30 (27.8%) were women. Twenty-seven (25.0%) eyes were diagnosed with diffuse retinal thickness, 65 (60.2%) with cystoid macular edema, and 16 (14.8%) with serous retinal detachment.

Table 1 shows an overview of the main demographic and clinical characteristics of the study sample. There were no significant differences in any of the baseline variables between the early-switch and late-switch groups.

**Table 1:** Baseline demographic and clinical characteristics

Characteristic	Overall (*n* = 108)	Early-switch (*n* = 32)	Late-switch (*n* = 76)	*P*^a^
Age, years				
Mean (SD)	67.6 (8.9)	67.7 (7.7)	67.5(9.4)	0.9651
95% CI	65.9 to 69.3	64.9 to 70.5	65.3 to 69.6	
HbA1c (%)				
Mean (SD)	7.3 (1.2)	7.4 (1.4)	7.3 (1.1)	0.9142
95% CI	7.1 to 7.6	6.9 to 7.9	7.1 to 7.6	
Sex, *n* (%)				
Man	78 (72.2)	26 (81.2)	52 (68.4)	0.1761^b^
Woman	30 (27.8)	6 (18.8)	24 (31.6)	
Eye, *n* (%)				
Right	48 (44.4)	12 (37.5)	36 (47.4)	0.3482^b^
Left	60 (55.6)	20 (62.5)	40 (52.6)	
Type of DM, *n* (%)				
Type 1	2 (1.9)	0 (0.0)	2 (2.6)	0.3565^b^
Type 2	106 (98.1%)	32 (100.0)	74 (97.4)	
Length DM, months				
Mean (SD)	16.8 (7.3)	17.8 (6.5)	16.4 (7.6)	0.1505
95% CI	15.4 to 18.2	15.4 to 20.1	14.6 to 18.1	
Type of DME, *n* (%)				
DRT	27 (25.0)	9 (28.1)	18 (23.7)	
CME	65 (60.2)	16 (50.0)	49 (64.5)	0.2887^b^
SRD	16 (14.8)	7 (21.9)	9 (11.8)	
NRD, *n* (%)				
Yes	9 (8.3)	4 (12.5)	5 (6.6)	0.3116^b^
No	99 (91.7)	28 (87.5)	71 (93.4)	
Visual acuity*				
Mean (SD)	0,32 (0,23)	0.35 (0.27)	0.30 (0.21)	0.7055
95% CI	0.27 to 0.36	0.25 to 0.44	0.26 to 0.35	
CRT, µm				
Mean (SD)	431.3 (115.4)	434.0 (130.5)	430.1 (109.5)	0.8217
95% CI	409. to 453.3	387.0 to 481.1	405.1 to 455.2	
SCT, µm				
Mean (SD)	210.74 (68.4)	216.4 (82.7)	207.8 (61.8)	0.5927
95% CI	197.3 to 223.4	186.6 to 246.2	193.7 to 222.0	
Type of anti-VEGF, I (%)**				
Bevacizumab	18 (16.7)	7 (21.9)	11 (14.5)	
Ranibizumab	55 (50.9)	8 (25.0)	47 (61.8)	
Aflibercept	68 (63.0)	19 (59.4)	48 (63.2)	0.0658^b^

*SD* standard deviation, *CI* confidence interval, *DME* diabetic macular edema, *DRT* diffuse retinal thickness, *CME* cystoid macular edema, *SRD* serous retinal detachment, *NRD* neuroretinal detachment, *CRT* central retinal thickness, *SCT* subfoveal choroidal thickness, *NA* not applicable, *anti-VEGF* vascular endothelial growth factor inhibitor

^a^Mann-Whitney *U* test (between naïve and non-naïve patients)

^b^Chi-square test

^*^Decimal scale

^**^The sum total may be greater than 100%, as some eyes received more than one anti-VEGF

### Cost analysis

The median (Interquartile range) cost was significantly lower in the early-switch (€2724.3; IqR: €1774.0 to €3712.3) than in the late-switch group (€5864.0; IqR: €4384.5 to €7763.6) (Hodges-Lehmann median difference: − 3057.8; 95% CI: 2406.4 to €3928.4, *p* < 0.0001).

An overview of the study costs is shown in Table 2.

**Table 2:** Cost effectiveness ratio and incremental cost effectiveness ratio analysis considering the total direct (with injection and without injection) in the cost of the Spanish Public Health System

	Cost with injection						
	Early-switch*			Late-switch*			
	Cost**	Outcome (numerical)^1^	CER	Cost**	Outcome (numerical)^1^	CER	ICER
BCVA gain ≥ 0.1 at any	2724.3	0.719	3789.0	5864.0	0.487	12,041.1	13,533.2
BCVA gain ≥ 0.1 at month 12	2724.3	0.438	6219.9	5864.0	0.316	18,557.0	25,735.2
BCVA gain ≥ 0.2 at any	2724.3	0.594	4586.4	5864.0	0.250	23,456.0	9127.0
BCVA gain ≥ 0.2 at month 12	2724.3	0.344	7919.5	5864.0	0.132	44,424.2	14,809.9
Per median BCVA gained^†^	2724.3	0.28	9729.6	5864.0	0.17	34,494.1	28,542.7
CRT ≤ 250 µm at month 12	2724.3	0.438	6219.9	5864.0	0.500	11,728	50,640.3

*ICER* = (cost in early-switch–cost in late-switch group)/(effectiveness in early-switch group–effectiveness in late switch group)

*CER* cost-effectiveness ratio, *ICER* incremental cost-effectiveness ratio

^*^The median total cost was used in the analysis

^**^Euros

^1^Proportion of eyes who achieved a specific BCVA gain or CRT reduction

^†^Difference between baseline and month 12 visit

The CER of BCVA improvement ≥ 0.1 at month 12 was € 3,789.0 in the early-switch group and € 12,041.1 in the late-switch one. Early-switch group showed a lower CER in all the outcome measured (Table 2).

Regarding ICER, late-switch group was associated with an incremental cost of €25,735.2 and €13,533.2 for achieving a BCVA improvement ≥ 0.1 at month 12 and at any of the time-point measured, respectively (Table 2).

### Changes in BCVA

At month 12, 38 (35.2%) eyes achieved a BCVA improvement ≥ 0.1. The probability of achieving a BCVA improvement ≥ 0.2 at any time-point and at month 12 was significantly greater in the early-switch group than in the late-switch one (*p* = 0.0060 and 0.0224, respectively).

The probability of achieving a specific improvement in BCVA is shown in Table 3.

**Table 3:** Probability of achieving a specific clinical outcome

	Overall		Early-switch		Late-switch		IR difference	95% CI	*p*
	IR	95%CI	IR	95%CI	IR	95%CI			
BCVA gain ≥ 0.1 at any	0.556	0.424 to 0.715	0.719	0.456 to 1.08	0.487	0.343 to 0.671	0.231	− 0.08 to 0.540	0.1398
BCVA gain ≥ 0.1 at m12	0.352	0.249 to 0.483	0.438	0.239 to 0.734	0.316	0.202 to 0.470	0.121	− 0.123 to 0.367	0.3302
BCVA gain ≥ 0.2 at any	0.352	0.249 to 0.483	0.594	0.358 to 0.927	0.250	0.151 to 0.390	0.344	0.099 to 0.589	0.0060
BCVA gain ≥ 0.2 at M12	0.194	0.120 to 0.297	0.344	0.172 to 0.615	0.132	0.063 to 0.242	0.212	0.030 to 0.394	0.0224
CRT ≤ 250 µm at month 12	0.482	0.360 to 0.631	0.438	0.239 to 0.734	0.500	0.354 to 0.686	− 0.063	− 0.349 to 0.224	0.6691

*IR* incidence rate, *95%CI* 95% confidence interval

BCVA was significantly greater in the early-switch group than in the late-switch one at month 8 (mean difference: 0.11; 95% CI: 0.0 to 0.21, *p* = 0.0311) and month 12 (mean difference: 0.13; 95% CI: 0.04 to 0.23, *p* = 0.0078). As compared to baseline, BCVA significantly improved in the early-switch group (*p* = 0.0094, repeated ANOVA test) but not in the late-switch group (*p* = 0.1617).

The Cochran–Mantel–Haenszel odds ratio of achieving a BCVA gain ≥ 0.1 at any time-point measured was 2.88 (95% CI: 1.12 to 6.82; *p* = 0.0254) (Table 4).

**Table 4:** Relationship between probability of achieving a certain functional or anatomic outcome and treatment-group. Late-switch group was used as reference in all the variables. Statistical analysis was done by the Cochran–Mantel–Haenszel odds ratio test

Outcome	Odds ratio*	95% CI	*p*
BCVA gain ≥ 0.1 at month 12	1.70	0.71 to 4.03	0.2256
BCVA gain ≥ 0.1 at any time-point measured	2.88	1.12 to 6.82	0.0254
BCVA gain ≥ 0.2 at month 12	3.58	1.30 to 9.87	0.0121
BCVA gain ≥ 0.2 at any time-point measured	4.35	1.79 to 10.59	0.0009
CRT ≤ 250 µm at month 12	0.81	0.35 to 1.88	0.6222

*CI* confidence interval, *BCVA* best corrected visual acuity, *CRT* Central retinal thickness

^*^Odds ratio was adjusted by type of edema

### Changes in CRT and SCT

At month 12, 52 (48.1) eyes had achieved a CRT ≤ 250 μm, without significant differences between the early-switch (14/32 eyes, 43.8%) and late-switch groups (38/76, 50.0%), *p* = 0.6691 (Table 3).

In the early-switch group, DEX-I significantly reduced CRT from 431.3 ± 115.5 µm at baseline to 281.7 ± 69.2 µm, 373.9 ± 126.5 µm, 358.2 ± 108.2 µm, and 269.3 ± 66.2 µm at months 2, 4, 8, and 12, respectively (*p* < 0.001, repeated measures ANOVA and the Greenhouse–Geisser correction).

Similarly, in the late switch group, mean CRT was significantly reduced from 430.1 ± 130.1 µm at baseline to 276.6 ± 66.6 µm, 380.2 ± 134.8 µm, 361.6 ± 113.4 µm, and 268.6 ± 66.1 µm at months 2, 4, 8, and 12, respectively (*p* < 0.001, repeated measures ANOVA and the Greenhouse–Geisser correction).

**Fig. 1:**
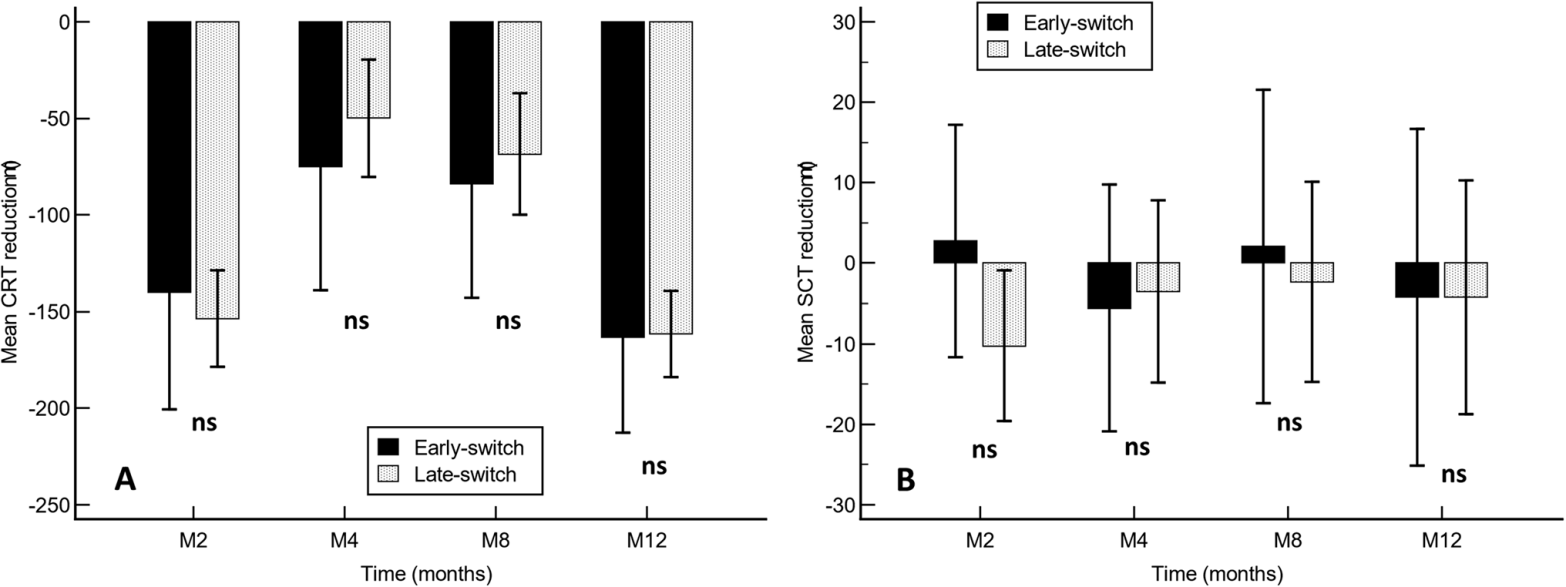
Mean change in central retinal thickness (**A**) and subfoveal choroidal thickness (**B**) in early-switch and late-switch groups. Vertical bars represent 95% confidence interval. Ns, not significant; CRT, central retinal thickness; SCT, subfoveal choroidal thickness

Mean changes in CRT and SCT were similar in early-switch and late switch groups (Fig. 1 A and B, respectively). As compared to baseline, the mean (95% CI) CRT reduction was − 163.1 (− 212.5 to − 113.7) µm and − 161.6 (− 183.8 to − 139.3) µm in the early-switch and late-switch groups, respectively, *p* = 0.9463.

## Discussion

Public health services have to cope with unlimited demand with limited resources. Further, the imbalance between demand and supply is assumed to be deteriorating as the population ages, new technologies appear, and expectations rise [28]. For everything mentioned so far, it is extremely important to identify cost effective treatments.

DME represent an increasing economic burden to health systems not only due to direct costs but also indirect ones, such as reduced income or an increased need for social support as vision worsens [29]. In addition, it has been estimated that the total cost per patient with DME represents 30% more than that of DM patients without DME [30].

In recent years, the introduction of new therapies for DME has led to a paradigm shift in its clinical management. Furthermore, the choice of treatment options for DME depend critically on individual patient clinical characteristics [9].

Using real-world data, we evaluated the direct medical cost of two different treatment strategies in patients with DME, namely, early-switch (eyes who did not adequately respond to 3-monthly injections of anti-VEGF were switched to DEX-i) versus late-switch (eyes who did not respond properly to 3-monthly injections of anti-VEGF and received an extending initial treatment of 3-monthly anti-VEGF injections up to 6 months, before switching to DEX-i).

According to the results of the current study, early-switch strategy was associated with a total cost saving of € 3057.8. Additionally, late-switch was associated with an ICER of €25,735.2 and €13,533.2 for achieving a BCVA improvement ≥ 0.1 at month 12 and at any of the time-point measured, respectively.

As far as we know, this is the first study evaluating the cost of extending the anti-VEGF treatment dose before switching to DEX-i.

In a previous paper published by our group, we evaluated the cost of extending the anti-VEGF treatment from 3 to 6 monthly injections [25]. This study revealed the incremental costs of extending the anti-VEGF dose in central-involved DME patients who initially did not respond adequately to treatment.

Although extending the anti-VEGF dose from 3 to 6 monthly injections has been associated with better outcomes [14, 31], this option involves treating all patients, regardless the final outcome.

In fact, extending the anti-VEGF initial treatment from 3 to 6 intravitreal injections has entailed a cost increased of €5882.77, €10,091.03, and €10,198.59 per additional responder patient (3-month nonresponders and 6-month responders) to aflibercept, ranibizumab and bevacizumab, respectively [25].

The economic impact of the introduction of DEX-i for the treatment of DME on the Spanish National Health System was estimated using a 3-year budget impact model [32]. This study concluded that the introduction of DEX-i in the Spanish market resulted in significant cost savings, due mainly to fewer injections with DEX-i [32].

In the current study, switching to DEX-i was associated better functional outcomes in the early-switch group and with better anatomic outcomes in both early-switch and late switch groups.

Real-world data have suggested that in DME eyes who did not adequately respond to anti-VEGF, switching to DEX-i is a feasible and safe strategy [19, 20, 33–37].

The results of these studies point in the same general direction, indicating that in patients who did not adequately respond to anti-VEGF therapy, switching to DEX-i provides better functional and anatomical outcomes [19, 20, 33–36].

The current study found a significant improvement in BCVA in the early-switch group, but not in the late-switch one. In DME patients, there seems to be an association between BCVA improvement and external limiting membrane (ELM) integrity [38, 39]. This may suggest a significant relationship between ELM integrity and photoreceptor cell bodies status, which may be a sign of advanced photoreceptor damage [38, 39]. DEX-i significantly improved ELM integrity, which was correlated with a significant with the upturn in BCVA [40]. Since the baseline integrity of ELM was associated with better functional outcomes, it makes sense to assume that an early recovery of these structures would be associated with better functional outcomes [41]. These findings may explain the differences in functional outcomes between the early switch and late switch groups observed in our study. However, further studies are needed to confirm this assumption.

Regarding the anatomic outcomes, our study found a significant reduction in CRT, without differences between groups. These findings are in agreement with the current evidence [19, 20, 33–36].

Our study has some limitation that need to be taken into account when interpreting the results. Although this study used data from a cohort of patients from three centers in its model, the number of eyes and the geographical distribution of the sample may limit the generalization of the results obtained. Nevertheless, as an important strength of our study, it collects real-world data, which are more representative of the unselected population that we usually attend in our daily clinical practice [42]. Additionally, our study did not evaluate direct non-medical-related costs (i.e. home healthcare and social services), patient transportation, or other incidentals, to establish economic parameters.

## Conclusions

In DME patients who do not adequately respond to anti-VEGF switch to DEX-i at early stages (after the first 3-monthly injections) was found to be more cost-effective than extending the initial treatment to 6-monthly injections of anti-VEGF. Additionally, DEX-i significantly improved the anatomic outcomes in difficult cases that have failed to respond to previous anti-VEGF therapies. However, the functional outcomes were significantly improved only in the early-switch group, which speak in favor of switching to DEX-i as soon as possible.

Although anti-VEGF are currently considered as the first line therapy in DME patients, many patients do not adequately respond to them. Therefore, further studies will be necessary to determine the most cost-effective treatment according to the patient’s profile.

## Data Availability

Data not here published are obtainable on reasonable request from the corresponding author.
